# Development of a Personal Integrated Environmental Monitoring System

**DOI:** 10.3390/s141122065

**Published:** 2014-11-20

**Authors:** Man Sing Wong, Tsan Pong Yip, Esmond Mok

**Affiliations:** Department of Land Surveying and Geo-Informatics, The Hong Kong Polytechnic University, Hong Kong; E-Mails: tpwyip@gmail.com (T.P.Y.); esmond.mok@polyu.edu.hk (E.M.)

**Keywords:** Android mobile application, environmental monitoring system, global positioning system, low-cost sensor

## Abstract

Environmental pollution in the urban areas of Hong Kong has become a serious public issue but most urban inhabitants have no means of judging their own living environment in terms of dangerous threshold and overall livability. Currently there exist many low-cost sensors such as ultra-violet, temperature and air quality sensors that provide reasonably accurate data quality. In this paper, the development and evaluation of Integrated Environmental Monitoring System (IEMS) are illustrated. This system consists of three components: (i) position determination and sensor data collection for real-time geospatial-based environmental monitoring; (ii) on-site data communication and visualization with the aid of an Android-based application; and (iii) data analysis on a web server. This system has shown to be working well during field tests in a bus journey and a construction site. It provides an effective service platform for collecting environmental data in near real-time, and raises the public awareness of environmental quality in micro-environments.

## 1. Introduction

Urban areas are growing progressively in many metropolitan cities, and thus more urban inhabitants are subjected to the compromising burden of living in a polluted environment. It is well recognized that excessive exposures to heat, ultra-violet (UV) radiation, noise, and air pollution may result in injury, chronic illness, permanent disability or even death. In construction sites, the different hierarchy of personnel who carry out prolonged tasks under direct sunshine may cause sunburn, heat exhaustion, and even heat stroke. Moreover, long-period exposure to a high decibel environment generated by machines and construction plants may cause hearing loss, and excessive exposure in a polluted air environment may cause lung-related diseases. Monitoring the changes of these major environmental factors is therefore critical for controlling, regulating and mitigating environmental pollution. Many developed countries such as United States and Canada have already provided standard indices related to environment quality to the general public. In Hong Kong, the Hong Kong Observatory (HKO) and the Hong Kong Environmental Protection Department (HKEPD) have released the UV index [1] and Air Quality Health Index (AQHI) [2] to the public in regular basis. These indices provide a warning mechanism and instruction to those inhabitants who are sensitive or vulnerable to environment-related health problems. However, these indices are spatially restricted by discrete stations distribution. Due to the high cost and complexity of station-based environmental monitoring systems, there is a necessity to develop a portable and low-cost integrated environmental monitoring system.

Mobile environmental sensing is the integration of different environmental detection sensors with data communication device into one system, in which the data acquired can be used for further processing and visualization [3,4]. There are several environmental sensing projects conducted particularly for air quality monitoring, e.g., Rudman *et al.* [5] implemented a project “THE eGS SYSTEM” on measuring air quality using a carbon monoxide (CO) sensor associated with GPS receiver. The CO values were displayed on a mobile tablet. N-SMARTS [6] proposed a COTS platform for integrating CO, NO_x_ sensors with GPS-embedded phone into a single pack, using Bluetooth as the communication tool between sensors and smartphones. Area's Immediate Reading (AIR), a public social experiment in New York, developed a Preemptive Media's portable air monitoring devices to monitor their neighborhood and pinpoint air pollution and fossil fuel burning hotspots [7]. Mead *et al.* [8] and Williams *et al.* [9] also developed low-cost portable devices for measuring air quality and ozone, respectively. NoxDroid [10] was a project to monitor air quality in urban cities using a small mobile sensor device mounted on bicycles equipped with smartphones. The sensor device could be attached to the handlebar of bicycles. The device adopted an MQ-135 Air Quality sensor, which measured NOx, NH_3_, alcohol, benzene, smoke and carbon dioxide, and was connected via USB cable to a smartphone. The data and their associated positioning information were uploaded to a web server for further processing. However, this air quality sensor requires high energy consumption in its small heater, thus the battery life is comparatively short. SiNOxSense [11] works like the NoxDroid, but it is wearable and able to provide location information from the network provider. Although numerous environmental sensing systems have been developed, their primary objectives are focused on air quality monitoring.

Currently, only a few projects integrate multiple sensors into one unified system. For example, Common Sense [12,13] developed a portable handheld device that measured CO, NO_x_, O_3_, temperature and humidity data associated with GPS location. These data were uploaded to a database server through GPRS [13]. Kanjo *et al.* [14] developed a monitoring system named “MobGeoSen”; it was consisted of a default sound level sensor in a mobile phone, environmental sensors with data logger, a GPS receiver, and Bluetooth communication module. However, these sensors and communication devices were not integrated into a single unit.

Although some low-cost environmental sensing devices are available on the market, with the escalating demand and use of smartphones, there is an urgent need to develop a personal environmental monitoring system integrating low-cost sensors, mobile application on smartphones, and GPS positioning.

## 2. System Design and Implementation

### 2.1. System Overview

**Figure 1.**
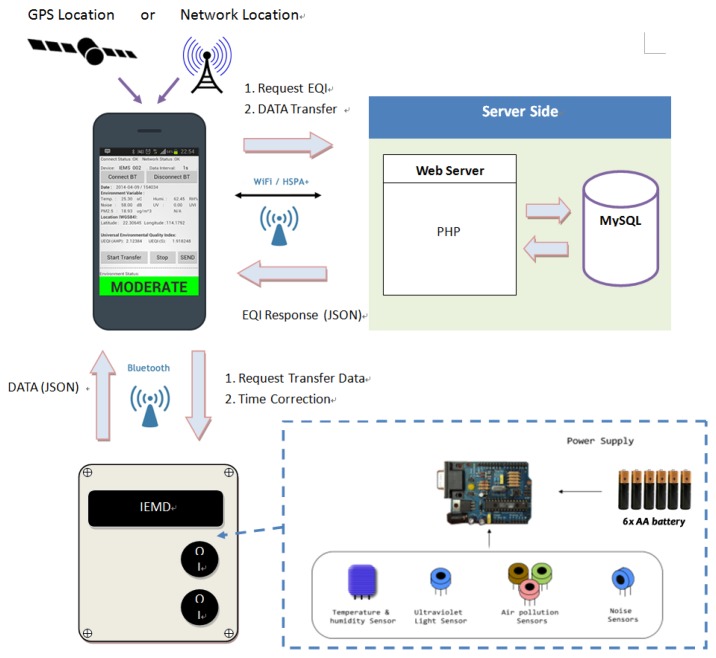
System overview of IEMS.

This paper demonstrates an Integrated Environmental Monitoring System (IEMS) for sensing the micro-environment. Figure 1 shows the system overview of IEMS. It consists of three major components: (i) an Integrated Environmental Monitoring Device (IEMD); (ii) a handheld Remote Control Panel (RCP) based on Android application; and (iii) a web server.

The IEMD is an integrated platform for environmental sensing which is equipped with a microcontroller, wireless communication module, and environmental sensors including temperature, humidity, UV, sound level, and air quality. RCP is a portable remote control interface for the IEMD, and it is used for device control, data communication between device and web server, and positioning. The web application includes web server and web interface which is constructed based on a PHP compliant Apache web server with MYSQL database. The web server provides a centralized data storage interface for data communication to RCP, data analysis and visualization.

Acquired environmental data on the IEMD are transferred to the RCP through Bluetooth communication. Environmental data associated with positioning information provided by the smartphone are then transmitted to web server for data analysis, via 3G or Wi-Fi in real-time. Once data analysis is completed, the web server will provide a response message including the environmental quality and other related information, e.g., precaution measures, back to the RCP. All the environmental and positioning data, as well as the processed data will be stored in the web server.

### 2.2. Integrated Environmental Monitoring Device (IEMD)

**Figure 2.**
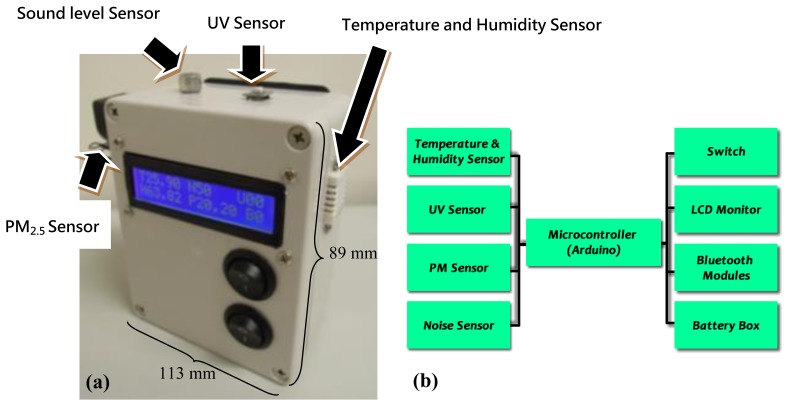
(**a**) Exterior view of IEMD; (**b**) interior view of IEMD.

IEMD is a portable, compact, battery powered long-lasting device, consisting of several components including the microcontroller, environmental sensors and wireless communication module (Figure 2). The power for the device is supplied by six AA alkaline batteries. Each of these components is described in the following section.

#### 2.2.1. Processor Module

**Figure 3.**
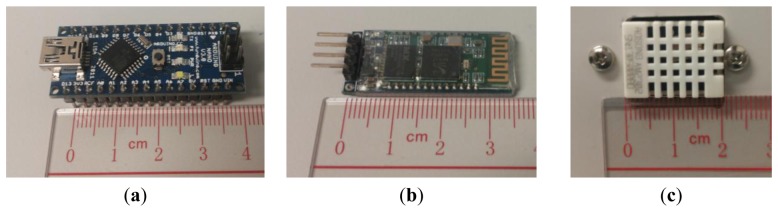
(**a**) Arduino nano board; (**b**) Bluetooth 2.0 module; (**c**) AM2302 digital temperature and relative humidity sensor module.

An Arduino nanoboard is used in IEMD, which is a single board microcontroller, consisting of an Atmel 8 bit ATmega328AVR microcontroller with other circuit components (Figure 3a). Android board embeds a 5 volt linear regulator for power source output and a 16 MHz crystal oscillator. It provides several pins that allow connecting other external components, and two of them support serial communication.

#### 2.2.2. Communication Module

HC-06 Bluetooth module is adopted for wireless communication between the IEMD and RCP (Figure 3b). Bluetooth has been recognized as an effective mode for short range data communication because it has relatively low power consumption and low-cost compared with Wi-Fi or GSM data transmission [15].

#### 2.2.3. Temperature and Humidity Module

The AM2302 digital temperature and relative humidity sensor module is embedded in the IEMD (Figure 3c). This module embeds a Negative Temperature Coefficient (NTC) thermistor temperature sensor, polymeric film humidity (capacitance type) sensor, and 16 bits analogue to digital convertor with serial ports for digital data communication. The NTC thermistor sensor is made up of a small semiconductor where the electrical resistance varies inverse proportionally to the temperature. The capacitive polymeric film humidity sensor is made of a substrate on which a humidity sensitive layer is in between two electrodes in order to measure the capacitance changes [16]. The AM2302 sensor is not only low-cost and small size, but it also has wide measurement range, long term stability and low power consumption. Its operating range of temperature is from −40 °C to 80 °C with 0.1 °C accuracy, and humidity can be measured in a range of 0%–100%.

**Figure 4.**
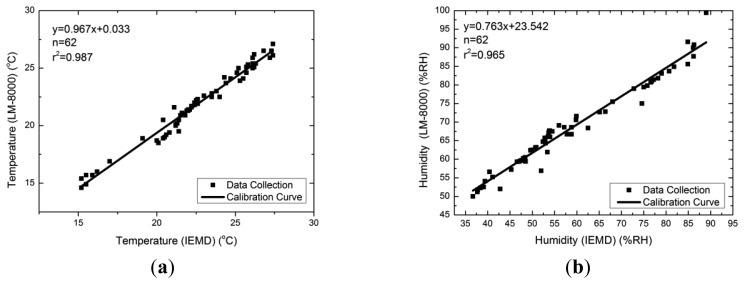
Calibration curve for (**a**) temperature sensor; (**b**) humidity sensor.

The AM2302 sensor was calibrated with an Environment Anemometer (LM-8000, LUTRON, Coopersburg, PA, USA) at a distance of 10 cm, in a non-air-conditioned room under long period observation. The temperature and humidity readings were recorded when the readings were stable. This normally takes two to five minutes after power on. The temperatures measured by the AM2302 are usually higher than those measured from the Environment Anemometer by an average of 0.7 °C, the measured humidity values from AM2302 are generally lower than those from the Environment Anemometer by an average of 9.54 RH%. Figure 4 shows the calibration curve of temperature and humidity readings.

#### 2.2.4. UV Sensor

**Figure 5.**
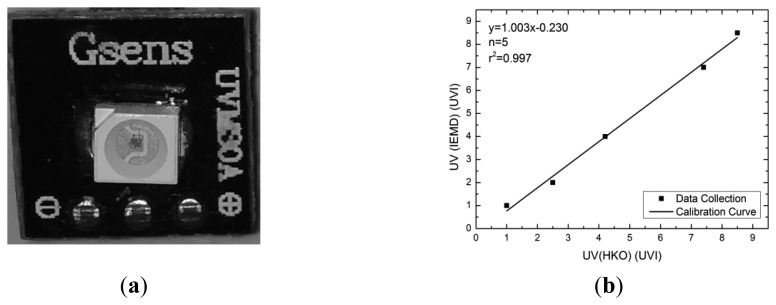
(**a**) UVM-30A sensor; (**b**) calibration curve for UV sensor.

The UVM-30A, manufactured by Guangzhou Logoele Electronics Technology Co. Ltd. (Guangzhou, China), selected as UV sensor and embedded in the IEMD, is small (9 × 9 × 10 mm), low-cost (approximately USD $6), and has a high response time speed (<5 s). The photodiode on the UVM-30A is a GUVA-S10GD. Referring to the datasheet on [17], the spectral sensitivity operational range is from 200 to 370 nm, which covers 62.5% range of UV-A and all ranges of UV-B light. The UV Index is an indicator to represent the strength of UV radiation that is directly proportional to the intensity of UV radiation. The relationship between the UV index and exposure level is described in [2]. According to the relationship between the UV index and the voltage from the datasheet, a performance test was conducted by comparing the UV levels from the HKO with the IEMD. The results show that these readings are in a linear relationship (Figure 5b).

#### 2.2.5. Sound Level Sensor

Noise-induced hearing loss results from high noise exposures over an extended period [18]. The noise exposures can be measured by the sound pressure level. Low-cost small noise pollution sensors are very rare on the market, but low-cost sound sensors are available. An electret condenser microphone and LM358 amplifier were used to measure the sound pressure level as noise pollution sensor in the IEMD.

This sensor converts sound from an analogue into a digital signal (voltage), but it does not directly represent the sound level. The sound level can then be calculated by the Root Mean Square (RMS) of the voltage signal in a period of time, it is expressed as in Equation (1):

(1) $$\text{Sound level}=\sqrt{\frac{\sum_{\text{i}=0}^{100}{\left(\text{Voltage reading}\right)}_{\text{i}}^{2}}{{\text{t}}_{\text{end}}-{\text{t}}_{\text{start}}}}$$

**Figure 6.**
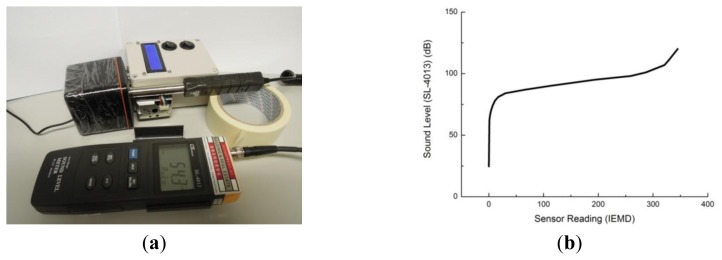
(**a**) Calibration setup for sound level sensor; (**b**) calibration curve for sound level sensor.

The sensor was calibrated with a sound level (dB) meter (LUTRON SL-4013 with standard IEC61672 type 2) with white noise generated by a computer, which produces a constant power spectral density independent of frequency. The setup is illustrated in Figure 6a. Figure 6b shows the relationship between sound levels and analogue readings from IEMD.

#### 2.2.6. Air Quality Sensor

Previous research has studied the use of a dust sensor to detect particulate matter (PM) concentrations [19,20]. In this study, a Sharp GP2Y1010AU0F dust sensor which is a compact, low-cost optical dust sensor, consisting of an infra-red emitting diode and a phototransistor, was used as air quality sensor in the system. It detects airborne particles using scattered light and is capable of detecting very fine particles. It is commonly used in air purifiers and air monitors.

**Figure 7.**
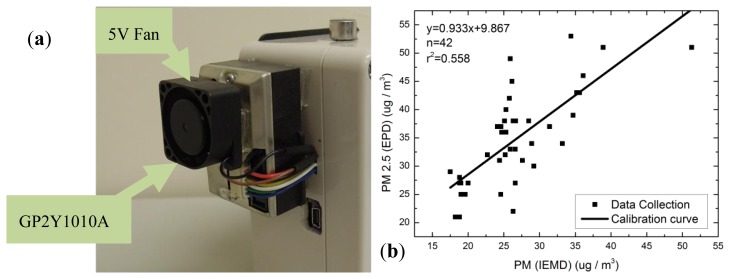

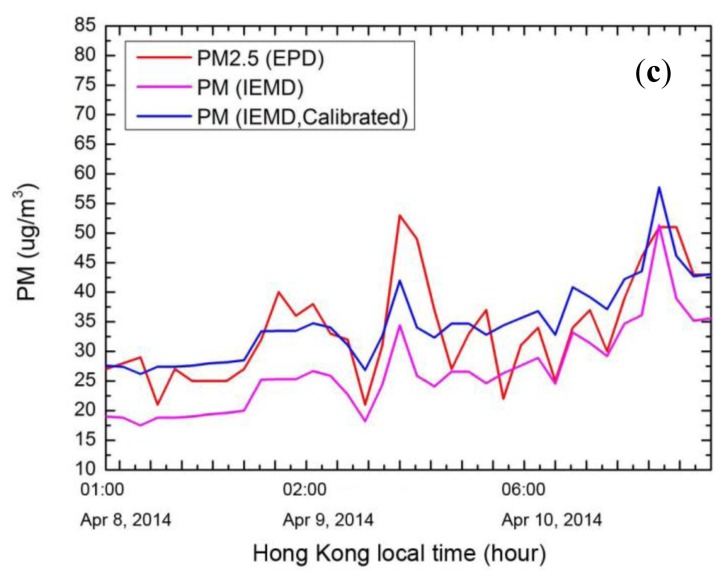
(**a**) PM_2.5_ sensor; (**b**) calibration curve for PM_2.5_ sensor; (**c**) comparison between EPD data with non-calibrated and calibrated IEMD data.

Since the HKEPD station is located in an access-restricted area, the IEMD can only be deployed in the nearest location, e.g., 50 m away at the same elevation. It is assumed that the PM concentrations would not vary significantly over a small distance. The calibration was performed for a couple of days using hourly averaged data. Figure 7b shows the calibration curve for the PM_2.5_ sensor. By comparing calibrated sensor readings with hourly HKEPD PM_2.5_ data, Figure 7c shows that the sensor is able to detect the PM_2.5_ in a moderate and reasonable accuracy.

#### 2.2.7. Hardware Programming

**Figure 8.**
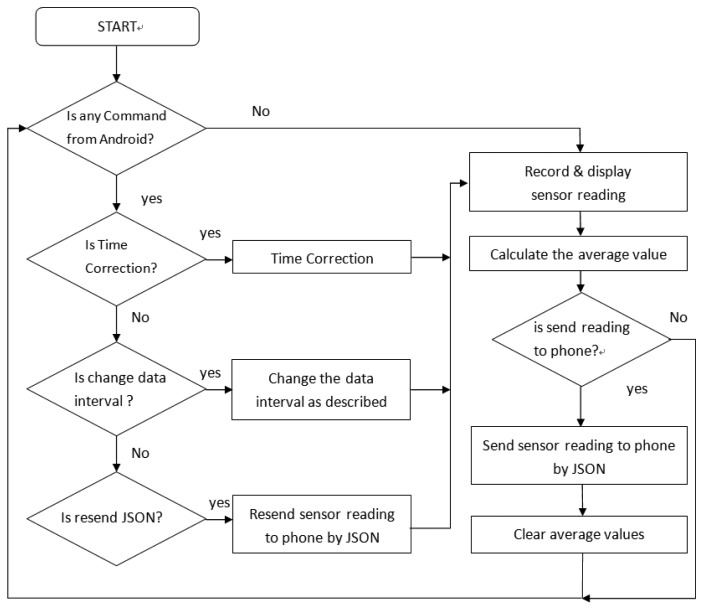
Flow chart of the IEMD.

The hardware control system of the IEMD is developed in an Arduino integrated development environment (IDE) programmed in C language. Figure 8 shows the flow chart of the hardware control system. When the device is switched on, the system will scan the incoming commands from the Android smartphone. The commands include time synchronization, data interval setting, and JavaScript Object Notation (JSON) resend. All commands are encoded in a JSON message before sending through Bluetooth communication. The time synchronization updates the time of device from the Android smartphone. The setting of the data interval allows users to define the period of data averaging, ranging from 1 s to 30 s. The JSON resend function is to re-transmit the previous data message as requested from the Android phone. Once the sensor readings are acquired, they will be averaged before being encoded into the JSON data message for data transmission. Data from the temperature and humidity sensor are sampled in every 2 s due to the response time; other sensors have 1 ms sampling intervals, with data averaged in every 1 s. The system also provides an interface for device control and device setting, including time correction, and interval setting for the data message broadcasting.

### 2.3. Android Application

**Figure 9.**
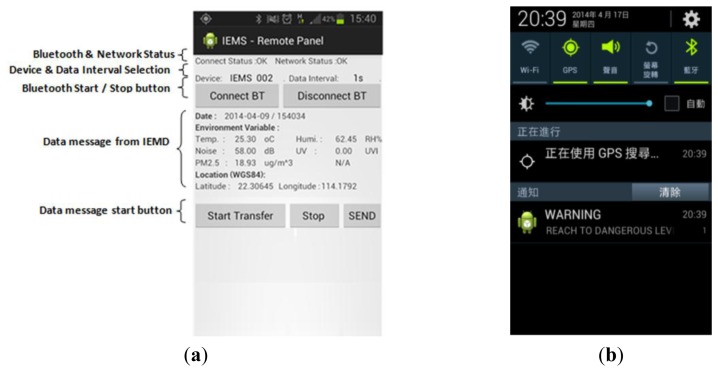
(**a**) User interface of RCP; (**b**) notification message in RCP.

The handheld Remote Control Panel (RCP) is an Android application which provides a user-friendly interface for device control (Figure 9a). In this study, the RCP is developed in Eclipse IDE with the Android developer tools of the Android Software Development Kit (SDK) [21]. It establishes an interface for IEMD device control, data exchange between the IEMD and web server, positioning, data visualization, as well as a notification service if the environmental readings exceed several thresholds.

The connection between the IEMD and RCP is established based on Bluetooth, all environmental data and their associated information including date, time, device name, device password and data sequence are encoded into data messages in JSON format before being transmitted to the RCP. Once a data message is received and verified, the RCP will acquire the positioning information from the Location Manager of the Android System using GPS, and the positioning information will be encoded into a JSON data message. Then the system will use the Apache HTTP Client to transmit the whole set of data from the RCP to the web server. Once the data analysis is completed, the web server will provide a response message including the environmental status and information back to the RCP for visualization. If the environment status condition exceeds a certain threshold, the system will trigger a notification to the user in the form of a warning message shown in the status bar (Figure 9b).

### 2.4. Web Application

The web application is mainly comprised of a series of web pages which embed the server-side scripting language PHP and the MYSQL database. The main functions of the web application are to receive the environmental data provided by the RCP, and to provide real-time data visualization as well as data analysis and visualization of archived data. The system provides a user-friendly web-based interface for data retrieval and analysis with an effective security approach.

Once the user logs into our web application system, a user session bounded with a token will be generated for user identification. The token has a length of 20 characters including the characters A–Z and 1–9, randomly generated from the system and saved in the database. When the user accesses the information from the web interface, the system will check whether the token stored in session matches with the one stored in the database. The system does not allow multiple accesses from different computing devices using same account.

**Figure 10.**
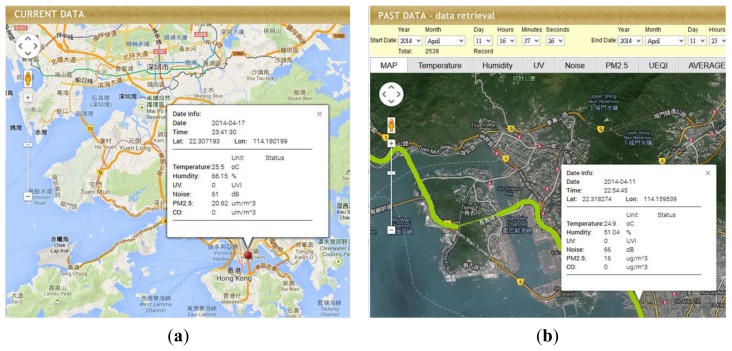

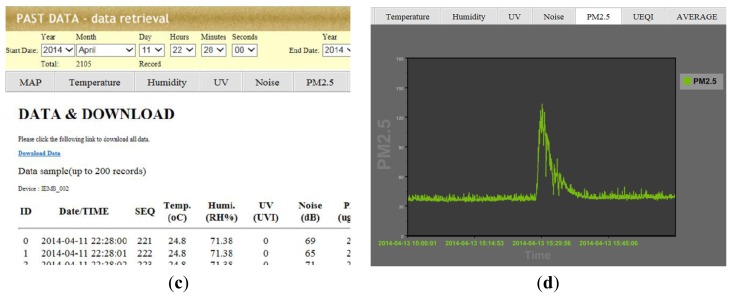
Web interface of (**a**) current data visualization; (**b**) archive data visualization; (**c**) data download page; (**d**) archive data of PM_2.5_ level.

Coupled with both Ajax and Google Map API, our system provides real-time monitoring and data visualization in the web interface (Figure 10a). The real-time data will be automatically uploaded and refreshed in the Google Map interface. Our system also provides archived data retrieval and visualization (Figure 10b). Once the user has selected the time period and pressed the “Search” button, the interface will retrieve data within the time period and display the data in the web interface. Selected data will be shown on Google Map, information window will pop-up which includes the date, time, location, and data measurements. Data summary, graphics and download functions are also provided in the system (Figure 10c and 10d).

## 3. Results and Discussion

### 3.1. Field Tests

Field tests were conducted in several locations for evaluating the performance and functionality of the system. A smartphone was placed in a small pocket on the shoulder harness of backpack, and an IEMD was hand-held while walking through several locations near campus such as some nearby road repair works and a bus terminal, on 10 April 2014, from 11:34 am to 12:06 am, and on 11 April 2014 from 10:24 pm to 10:30 pm. The IEMS was also tested in a bus journey on 11 April 2014 from 10:31 pm to 11:23 pm.

### 3.2. Sensor Reading

**Figure 11.**
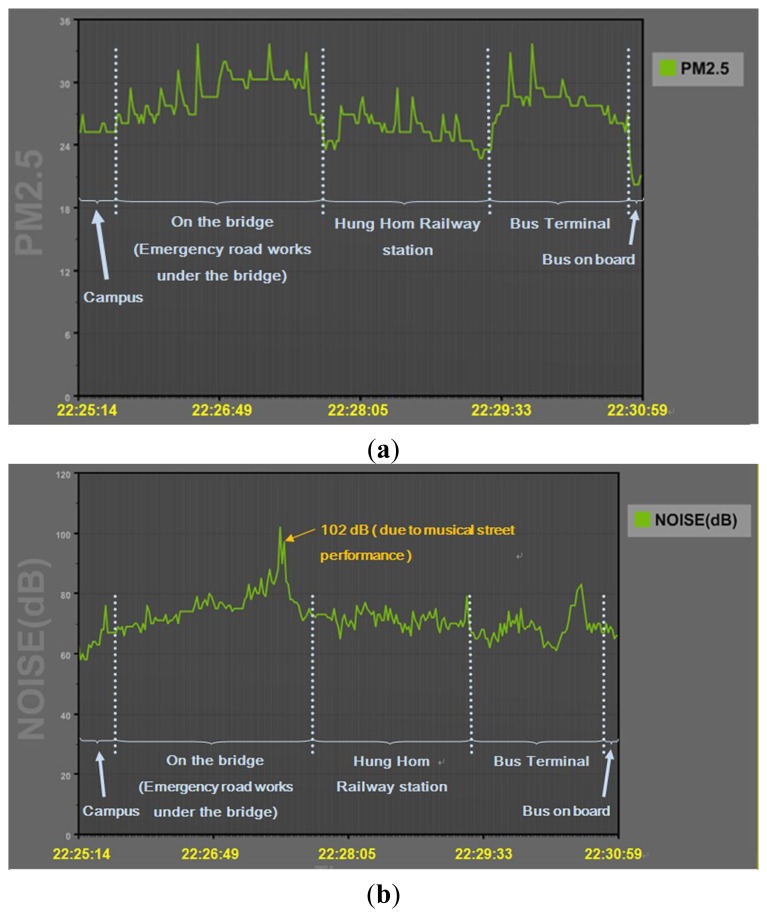
Field tests (**a**) PM_2.5_ level; (**b**) sound level.

**Figure 12.**
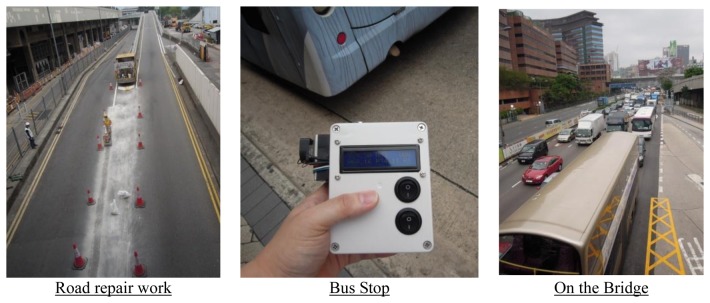
Locations of high PM_2.5_ concentrations during the field tests.

The designed system performed well during the field tests. All acquired environmental data were transferred to the server via an Android smartphone. The sensor outputs, representing the level of each environmental factor, were converted to values in standard units. This system was sensitive to detect *in-situ* environmental conditions during the field tests e.g., emergency road works. Figure 11a shows that the observed air quality values are higher when walking on the bridge and in the bus terminal where high PM_2.5_ concentrations are mainly due to the repair works and bus emission. Figure 11b shows that the sound level reaches a very high level at maximum 102 dB. Figure 12 shows these locations.

**Figure 13.**
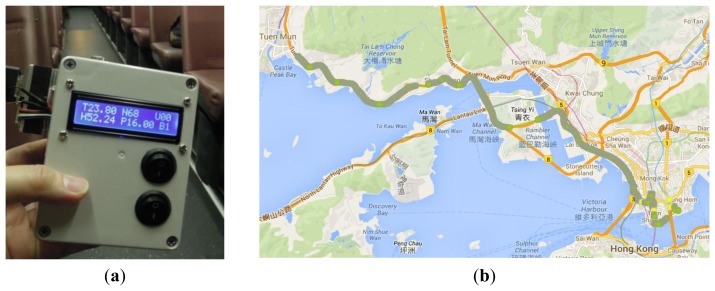
(**a**) IEMD measures the environment quality in bus compartment; (**b**) data collection in bus compartment.

The environmental quality in air-conditioning bus compartment was also tested (Figure 13a). In this study, an express bus route was selected, travelling from Kowloon to the New Territories and there was no stop on the highway (Figure 13b).

**Figure 14.**
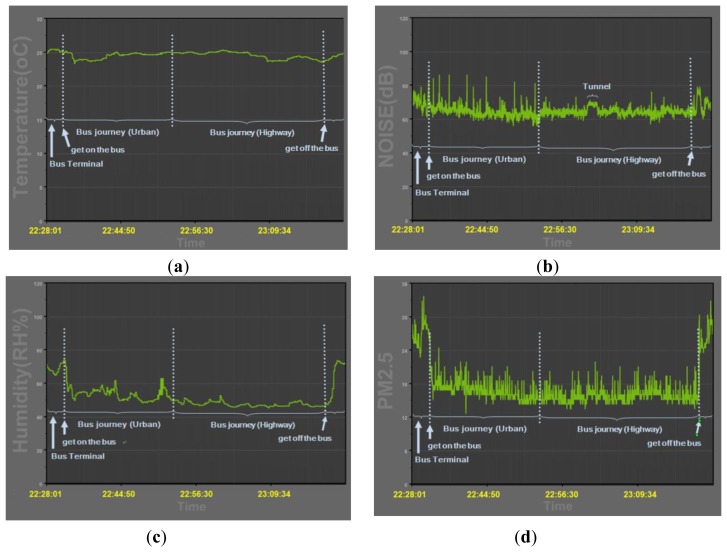
Achieve data of (**a**) temperature; (**b**) humidity; (**c**) sound level; (**d**) PM_2.5_ concentrations.

After boarding the bus, it was found that there were vast differences between the outdoor and indoor environments. Figure 14a–d shows the temperature, humidity, sound level and the PM_2.5_ concentrations on the bus.

The temperature was slightly increased when the number of passengers increased. It was also observed that the humidity increased rapidly during the passenger pick up and drop off periods as a result of the increased heat and water vapour from the outdoors. The PM_2.5_ values also dropped dramatically to a good level after boarding the bus. However, the sound level was in a range of 60 dB–70 dB in the bus compartment. The sound levels were even higher than 80 dB when the bus accelerated and decelerated. The sound levels were around 70 dB when the bus travelled through a tunnel, while the sound levels were reduced at around 63 dB when the bus was travelling on the highway.

**Figure 15.**
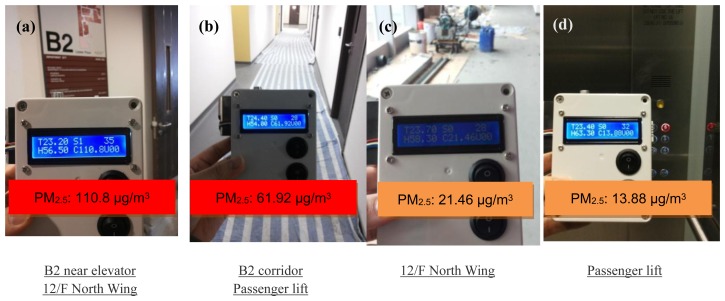
PM_2.5_ concentration in (**a**) basement; (**b**) basement corridor; (**c**) the 12/F; (**d**) the passenger lift.

The IEMS was also used to evaluate the air quality in a new campus building at the Hong Kong Polytechnic University during its construction phase (Figure 15). It showed that the air quality was not good in the basement where the construction works were still in progress, and the maximum PM_2.5_ concentration was reached to 110.8 μg/m^3^.

### 3.3. GPS Positioning

**Figure 16.**
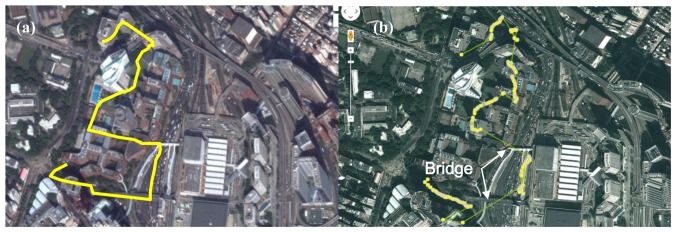
(**a**) Planned route for field test (yellow line); (**b**) data collection of field test (yellow points).

Smartphones usually use a relatively low-cost GPS chipset, where the performance of positioning accuracy is highly dependent on the number and position of GPS satellites. Figure 16a and b show that the position accuracy was not very good since the GPS signal might be blocked by surrounding buildings. It also shows that the GPS signal is totally blocked on the bridge due to the overhead cover.

### 3.4. Battery Life

Battery life is an important issue and not many systems can support long operating periods, thus, selecting low power consumption sensors is one of the key factors. Currently, the battery life of our IEMD version is approximately 30 h using six AA alkaline batteries. In previous research, the NoxDroid and SiNOxSense have short battery lives, and only last for 8 to 9 h, mainly due to the MQ-7 CO metal oxide sensor that has high energy consumption small heater, which is used for maintaining the sensing conditions during operation. There are two main factors controlling the battery life in our design: (i) display screen: the display screen consumes energy for lighting, thus a switch was designed. Users can switch off the screen when data are logging; (ii) Bluetooth 2.0: in our current design, Bluetooth is always enabled in our device. In future, Bluetooth 4.0 will be incorporated into the device which can reduce the power consumption [22], and with these changes the battery life is expected to be extended to more than 50 h.

## 4. Conclusions

Urban inhabitants are exposed to a wide variety of hazards, for example, heat, noise, UV radiation and air pollution. Excessive exposure to these hazards may result in injury, chronic illness, or even death. In recent years, environmental quality awareness has increased in our community, but the government locational-based environmental information may not be accurately represented the micro-environment situation, therefore, a real-time, automated, and integrated environmental monitoring system can significantly facilitate the health risk and hazard screening for inhabitants, especially the vulnerable groups.

This paper demonstrates a low-cost environmental sensing system with reasonable accuracy for immediate measurements of our living environment. This system consists of a portable device, an Android application and a web server. Different sensors for environmental monitoring (temperature, humidity, UV radiation, sound level, and air quality), positioning (GPS), communication (Bluetooth) and visualization (Android App) have been embedded along with a service platform to monitor possible environmental hazards. This study provides an effective service platform for environmental monitoring, and raises the public awareness about environmental quality in the micro-environment.

Smartphones consist of many built-in sensors including an accelerometer, gyroscope, and a camera. Current smartphones may also have environmental sensors such as barometers and thermometers as well as photometers. In the future, integration of these built-in sensors can enhance the performance and accuracy of our system. For example, the accelerometer and gyroscope can be integrated with GPS, providing better positioning. To reduce the size, the battery box which contains six AA alkaline batteries will be replaced by a thin polymer lithium battery. The IEMS can be used for a specific group of people for monitoring their micro-environments, one example is for outdoor workers. It is feasible to attach a compact device on the outdoor worker's clothing or hat for environmental quality monitoring. Safety officers can then assess the risk of outdoor work tasks via this system and take appropriate preventive measures for health and safety purposes.
